# Scalable, Lightweight, Integrated and Quick-to-Assemble (SLIQ) Hyperdrives for Functional Circuit Dissection

**DOI:** 10.3389/fncir.2017.00008

**Published:** 2017-02-13

**Authors:** Li Liang, Stefan N. Oline, Justin C. Kirk, Lukas Ian Schmitt, Robert W. Komorowski, Miguel Remondes, Michael M. Halassa

**Affiliations:** ^1^Department of Neuroscience and Physiology, New York University Neuroscience Institute, New York University Langone Medical Center, New YorkNY, USA; ^2^Picower Institute for Learning and Memory, Massachusetts Institute of Technology, CambridgeMA, USA; ^3^Instituto de Medicina Molecular, Faculdade de Medicina da Universidade de LisboaLisbon, Portugal; ^4^Center for Neural Science, New York University, New YorkNY, USA

**Keywords:** multi-electrode, microdrives, electrophysiology, single unit recording, adjustable electrode

## Abstract

Independently adjustable multielectrode arrays are routinely used to interrogate neuronal circuit function, enabling chronic *in vivo* monitoring of neuronal ensembles in freely behaving animals at a single-cell, single spike resolution. Despite the importance of this approach, its widespread use is limited by highly specialized design and fabrication methods. To address this, we have developed a Scalable, Lightweight, Integrated and Quick-to-assemble multielectrode array platform. This platform additionally integrates optical fibers with independently adjustable electrodes to allow simultaneous single unit recordings and circuit-specific optogenetic targeting and/or manipulation. In current designs, the fully assembled platforms are scalable from 2 to 32 microdrives, and yet range 1–3 g, light enough for small animals. Here, we describe the design process starting from intent in computer-aided design, parameter testing through finite element analysis and experimental means, and implementation of various applications across mice and rats. Combined, our methods may expand the utility of multielectrode recordings and their continued integration with other tools enabling functional dissection of intact neural circuits.

## Introduction

Experimental access to neural circuits has progressed rapidly with recent developments in optical and molecular tools. Combined with methods to monitor neural activity, these tools have enabled functional labeling of individual circuit elements (Xu et al., 2016; Ye et al., 2016). Importantly, neural recordings during well-controlled tasks provide an algorithmic readout for circuit function based on the spiking of individual neurons defined by connectivity, genetic identity, or both. While substantial progress continues with the use of primates (Graf and Andersen, 2015; Jazayeri and Shadlen, 2015; Kira et al., 2015; Lundqvist et al., 2016), rodents provide a complementary model given the relative ease of genetic and optical access (Dombeck et al., 2010; Brunton et al., 2013; O’Connor et al., 2013; Chen S.X. et al., 2015; Wimmer et al., 2015).

We wished to improve the process of accessing intact, optically identified neural circuits in freely behaving transgenic mice, however, currently available devices are either too heavy for small rodents, require expertise to make design alterations, or trade weight for mechanical stability. To overcome these challenges, we developed a Scalable, Lightweight, Integrated and Quick-to-assemble (SLIQ) multielectrode array platform that minimizes weight but maximizes structural stability. This platform employs a novel ‘microdrive’ design that enables adjustment of electrode position based on a spring and screw mechanism. Both the design of individual microdrives as well as that of its parent platform can be easily adjusted based on experimental needs, enabling functional circuit dissection across different models and species.

To optimize the sensitivity of these implants to stresses associated with electrode adjustment and freely moving behavior, we assessed the mechanical stability and electrode placement accuracy using structural modeling and measuring electrode displacement during adjustment. Finally, we provide validation our SLIQ hyperdrives by demonstrating use of these implants with stable recordings in a wide range of experimental designs, including both single unit recordings of genetically identified neuronal populations using optogenetic tagging in mice, and multisite local field potential recordings in rats.

## Materials and Methods

### Novel Microdrive Design Enables Scalability

In designing the SLIQ hyperdrive, we wished to maintain the ultra-light properties of the flexDrive (Voigts et al., 2013), along with the ability to precisely adjust electrode depth using a microscrew. However, we sought to implement drive scalability such that electrode number and drive body size could be quickly adjusted without the constant need to redesign the drive spring, and to add lateral stability to the head of the microscrew when adjusting electrode depth. We therefore took a systematic approach to creating a new drive mechanism, which resulted in our independently adjustable ‘microdrives.’ These can be added iteratively depending the number of desired independently adjustable electrodes, which merely requires adjustment of drive base and electrode interface board (EIB) holder dimensions. Once components have been prepared, assembly can take as little as 1 day, depending on the complexity of the preparation. Implants with higher numbers of tetrodes may take 2–3 days to complete, however, these larger hyperdrives provide substantial yields. Using 96 channel EIBs, we have recorded up to 45 units simultaneously in both the cortex and thalamus of mice.

### Computer-Aided Design of the Hyperdrive Body

**FIGURE 1:**
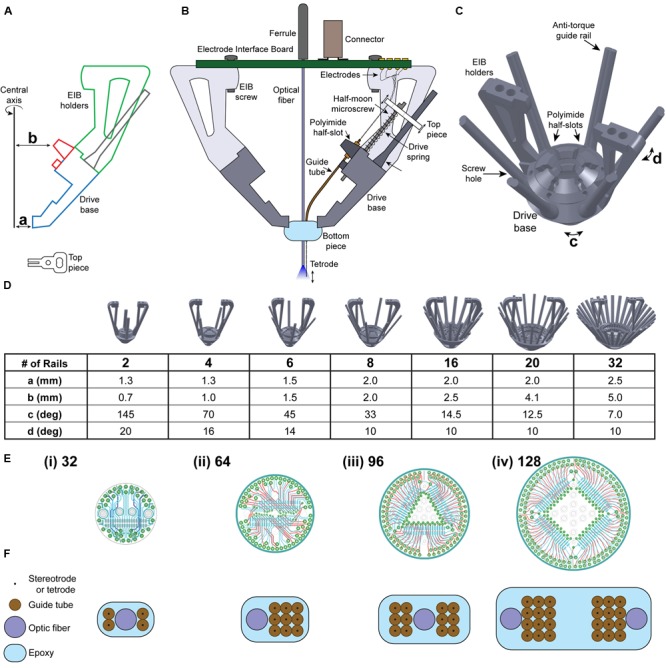
**Novel microdrive design enables scalability. (A)** Design of drive body and top piece using computer-aided design (CAD) software. Dimensions a, b, c, and d are adjusted depending on the number of guide rails needed. **(B)** A single microdrive unit, to be iteratively added to the SLIQ hyperdrive according to experimental needs. **(C)** Six-post SLIQ hyperdrive design, generated using SOLIDWORKS. **(D)** Demonstration of the scalability of this microdrive mechanism, with drive bases containing 2–32 guide rails. Table shows the values needed to scale drive design. **(E)** Schematics of (i) 32, (ii) 64, (iii) 96, and (iv) 128 channel electrode interface boards (EIBs). **(F)** Customizable bottom pieces and associated electrode array designs to scale with the number of microdrives.

We designed the SLIQ hyperdrive using SOLIDWORKS (Dassault Systemes, France), although other computer-aided design (CAD) software can also be used. The cross-section of the drive base, EIB holders, and polyimide half-slots are drawn on the front plane using the “Sketch” function (**Figure 1A**). Note that the distances from the central axis to the bottom of the drive base and to the polyimide half-slot (**Figure 1A**, a and b) are adjusted according to the number of microdrives desired. Suggested values are listed in the scalability table (**Figure 1D**). The drive body drawing is then revolved around the central axis for 360° using the “Revolved Boss/Base” function. Half-slots to hold the polyimide guide tubes are created by selecting the half-slot sketch (**Figure 1A**, red) and revolving it by the angle corresponding to the desired number of microslots (**Figure 1C**, angle c) to create one half-slot.

Using the “Circular Pattern” function, select both the central axis of revolution and the half-slot created in the previous step as the “Feature to Pattern,” and enter the number of half-slots to create. Similarly, the two EIB holders are created by selecting the EIB holder sketch (**Figure 1A**, green), and revolving it by the angle corresponding to the desired number of microdrives (**Figure 1C**, angle d). Then, using the “Circular Pattern” function, select the EIB holder created as the “Feature to Pattern” and create two holders. Microscrew receptacles are created by a new plane between two polyimide half-slots using the “Reference Geometry” function. Sketch the cross-sectional contours for one set of receptacles, polyimide half-slots, and guide rails onto the new plane. Then, use the “Extrude Boss/Base” function and select the anti-torque rail to extrude it upward for 10 mm and downward for 2 mm, and use the “Extrude Cut” function and select the polyimide half-slots and screw hole to extrude cut for 6 mm. Screw holes should be slightly smaller than the threading of the half moon screw (we use 0.41 mm thread minor, 0.58 mm thread diameter), and then carefully tapped with a drill bit. Conveniently, the drive body and number of microdrives can be quickly adapted to experimental needs by adjusting these four parameters. More detailed step-by-step CAD design procedures can be found in the Journal of Visual Experiments (Brunetti et al., 2014).

### Finite Element Analysis

To quickly evaluate many iterations of each implant component without the need to use a 3D printer, we turned to finite element analysis (FEA), a Solid Mechanics analysis method for structural evaluation of rigid objects under load. First, a 3D model is created of an object to be analyzed, and then redefined as a mesh consisting of thousands of nodes (finite elements) represented by systems of equations. These equations account for many material properties, such as density, rigidity, and yield strength, which is the load under which a material permanently deforms. The experimenter then assigns points where loads are applied, and FEA calculates an estimate of the stresses and deformations of the object. This output provides a useful first estimate for the design of the implant to then manufacture for empirical testing. Specifically, to evaluate the mechanical stability of the implant under load, we used the magnitude of stress across the 3D model in N/m^2^ and the displacement distance in mm. We performed FEA of our implant designs using the SOLIDWORKS Simulation software package (Dassault Systemes), and selected the material acrylonitrile butadiene styrene for analysis, which is the material we use for our 3D-printed components because of its strength and mild flexibility.

### Empirical Evaluation of Microdrive Stability

Since FEA of simulated materials does not fully take into account variations in loading due to screwdriver placement or the 3D printing process, it is important to validate conclusions *in situ*. We therefore empirically tested both microdrive stability and electrode placement accuracy. Top piece deflection angle (𝜃) theoretically affects electrode targeting accuracy by 2^∗^tan (Δ ^∗^ 𝜃). To assess the lateral movement of the top piece and guide rail during driving, we fixed the bottom of printed implants to a rigid surface with hot glue, and marked key locations of the top piece, anti-torque rail, and drive base for video analysis. We next drove the microscrew downward in quarter turns. Measurements were made by recording video at 30 frames per second using a 3MP MU300 digital camera (Amscope, Irvine, CA, USA). The screwdriver was kept parallel to the anti-torque guide rail during driving, and the locations of the marked points were determined using the Video Analysis function in LoggerPro (Vernier Software, Beaverton, Oregon, USA).

### Empirical Evaluation of Electrode Placement Accuracy

We assessed the relationship of top piece deformation with electrode targeting accuracy by devising a novel experimental setup consisting of fixing the SLIQ hyperdrive, and then driving a twisted tetrode (Stablohm 650, California Fine Wire Company, Grover Beach, CA, USA, 4 turns per cm) into a custom brain mimicking material. This material was made by mixing unflavored gelatin (E. D. Smith, Winona, ON, Canada) and water until its consistency after cooling was comparable to brain tissue, which was 3 g of gelatin per 250 mL of water. The gelatin was kept in place using a histology cube, and the front was cut out to allow for imaging. The implant was fixed onto a stereotaxic stage (David Kopf Instruments, Tujunga, CA, USA) using a custom stereotaxic adapter, made by affixing an EIB connector to a stereotaxic rod. To lower the electrode fixed to the top piece, we then used a custom screwdriver to drive the half-moon microscrew downward in quarter turns every 2 s for 15 turns, as noted above. Electrode bending was not assessed, as it is a function of the shear force exerted by the brain on the electrode, rather than top piece movement.

### Animals, Data Acquisition, and Analysis

All procedures adhered to the guidelines of the National Institutes of Health and were approved by the Institutional Animal Care and Use Committee at the New York University Langone Medical Center, the Committee on Animal Care at MIT, and the equivalent committee at IMM Lisbon. After implantation, electrodes were incrementally lowered into the target structure over the course of up to 2 weeks. Extracellular recordings were amplified, filtered, and acquired at 30–40 kHz, with the recording system and filters tailored to specific requirements across species and targets. For mouse LGN and TRN, recordings were band-pass filtered at 0.1 – 9,000 Hz, with online spike triggering via a Neuralynx Cheetah system, Neuralynx, Bozeman MT, and offline isolation using the MClust toolbox^1^. For mouse V1, recordings were high-pass filtered at 300 Hz, with offline spike triggering and isolation via Plexon Omniplex-A system (Plexon Inc., Dallas, TX, USA). For recordings in rats, spikes were extracted with a 300–6,000 Hz band-pass filter using an Open Ephys data acquisition setup run by Bonsai software, with offline spike triggering performed using a custom-made MATLAB script. Records shown of local field potentials are unfiltered.

### Viruses and Optical Fibers

For optogenetic tagging of identified auditory TRN units, we used FuGB2-pseudotyped retrograde lentiviruses (RG-LV) as described previously (Halassa et al., 2014). VGAT-Cre mice were injected with either DIO-ChR2-eYFP (for activation) or DIO-eNpHR3.0-eYFP (for inactivation) into the primary auditory thalamus [anterior-posterior (A-P), -3.15 mm; medial-lateral (M-L), -2.05 mm; dorsal-ventral (D-V), -3.15 mm; referenced to Bregma]. Titers were between 10^8^ and 10^9^ VG/ml, and 400–600 nL was injected at 50 nL/min with a Quintessential stereotactic injector (QSI, Stoelting, Wood Dale, IL, USA). Bottom pieces of these implants incorporated fixed optical fibers adjacent to the electrode array (200 μm diameter, 0.22 NA, Ocean Optics), which protruded 3.5 mm and were positioned immediately above the TRN (A-P, -2.00 mm; M-L, -2.00 mm; D-V, -2.80 mm; referenced to Bregma).

### Statistics

All data in Results are expressed as mean ± SD. Between groups comparisons were performed using ANOVAs to maintain estimates of effect size, which were reported as η^2^. Assumptions of sphericity were adjusted for with the Greenhouse-Geisser correction. Data analysis and plotting were performed with MATLAB (MathWorks) and Adobe Illustrator CC (Adobe Systems), and comparisons were calculated in SPSS Statistics (IBM).

## Results

### General Design of the SLIQ Multielectrode Array Platform

For a multi-electrode array to be used with freely moving small rodents such as the mouse, it must be designed within the boundaries of certain constraints (**Figure 1**). The total mass of the implant, including cement used to fix the implant to the skull, must be kept below three grams. Effort must therefore be taken to reduce excess material wherever possible when designing the implant structure, such as cutaways at areas not required for structural rigidity (**Figure 1A**). Despite this weight limit, the drive must be mechanically stable under the stresses of handling the animal while adjusting electrode position. The moving components for adjusting electrode depth must be rigid enough to maintain electrode position during behavioral tasks, yet allow for fine adjustment in increments of less than 50 μm. Finally, the implant design must preserve these properties while accommodating a wide range of implant sizes, electrode number and corresponding data acquisition channels, while allowing for varied organization of the multi-electrode array.

We therefore designed a novel approach to adjusting electrode depth with our ‘microdrives,’ which position electrodes using an assembly consisting of a top piece, a microscrew, a drive spring, a receptacle in the drive base for the screw to thread into, and an anti-torque guide rail (**Figure 1B**). The top piece holds the electrode in place along the axis of the microscrew, which then allows the user to raise and lower the electrode by turning the screw head with a custom screwdriver (an inverted half-moon screw glued inside a 16-gage needle). Using screws with a pitch of 150 μm allows quarter turns to advance electrodes in increments of roughly 37.5 μm (**Supplementary Table S1**). The drive spring ensures reliable electrode placement by pressing the upper surface of the top piece against the bottom of the screw head. Finally, the anti-torque guide rail minimizes lateral movement of the top piece during driving to ensure accurate electrode targeting. Importantly, these microdrives are assembled as individual units, and can be added iteratively to the drive body merely by adjusting two distances from the center axis to the drive body (*a* and *b*), and two radial angles corresponding to the angle between guide rails (*c*) and the thickness of the handles (*d*). **Figure 1C** shows a three-quarter view of a drive body designed for use with six microdrives, complete with a polyimide half-slot and guide rail for each microdrive. A key feature of the SLIQ hyperdrive is the ability to scale the number of microdrives as needed from 2 to 32, and here we provide suggested parameter values for several versions (**Figure 1D**).

Together, the EIB and connectors function to link electrodes with a data acquisition system. Each individual electrode must be pinned in place on the EIB with a gold pin. In general, larger implants with more microdrives and more electrodes will require EIBs that can accept more channels. We designed screw receptacles on the EIB holders to accept boards from manufacturers such as NeuraLynx and Open Ephys, however, our custom boards designed with EAGLE software are shown, and support from 32 to 128 channels (**Figure 1E**). Use of Omnetics connectors with the EIB ensures compatibility with widely used data acquisition systems from Blackrock Microsystems, Plexon, Open Ephys, and NeuraLynx. Ground and reference channels should be bridged to both a skull screw and an exterior shield for noise reduction. Additionally, if EEG recordings are desired, a single EIB channel should be reserved to connect to a second skull screw. Finally, similar to the flexDrive, many custom electrode array arrangements can be achieved by varying the positioning of polyimide guide tubes within the bottom piece (**Figure 1F**). This array is assembled by sequentially stacking layers of polyimide tubes held in place with cyanoacrylate glue, and the bundle is secured with epoxy. Accordingly, the same basic architecture can be used for multi-site recordings and single region, high density recordings. Depending on experimental needs, each guide tube and associated microdrive can shuttle a single electrode, a stereotrode, or a tetrode, though we have also had success double-loading stereotrodes onto a single microdrive.

### Evaluation of EIB Holder and Anti-torque Guide Rails Design With Finite Element Analysis

**FIGURE 2:**
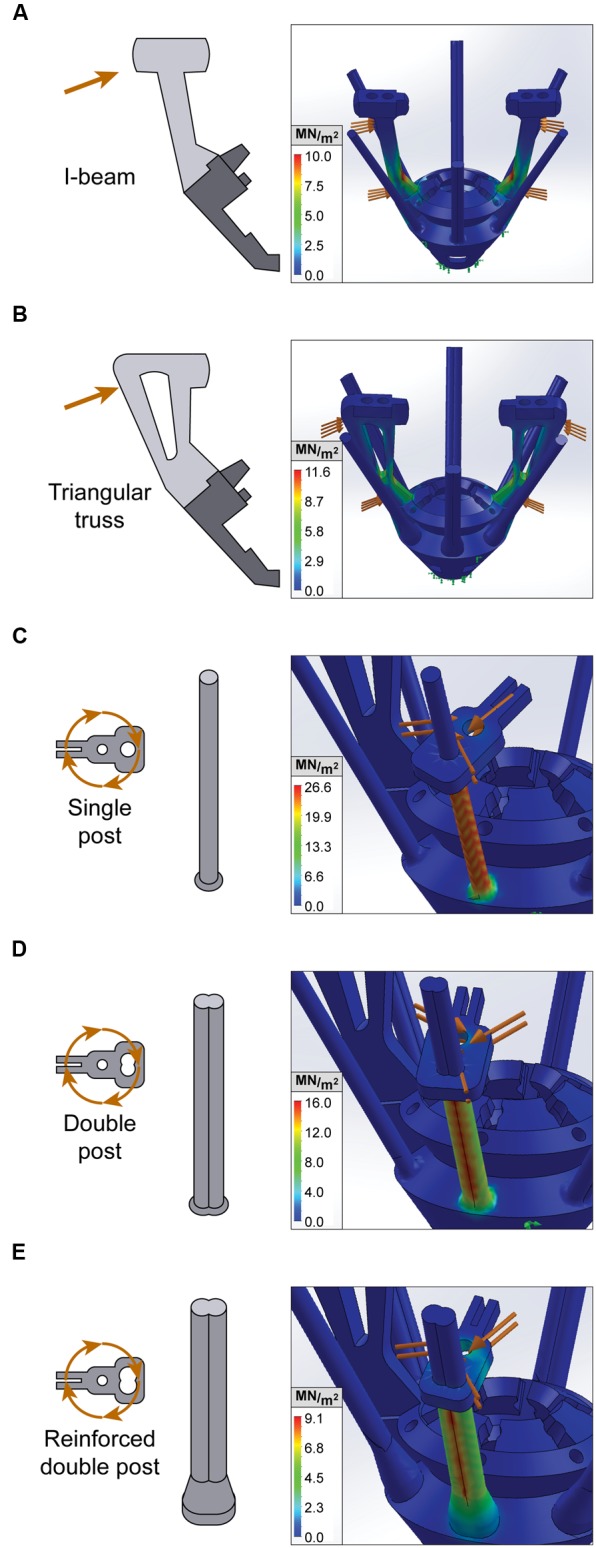
**Finite element analysis of EIB holders and anti-torque guide rails.** Stress tests for static loading conditions of constrained microdrive components. Orange arrows denote external loading, while green arrows denote points of fixation. von Mises stress is measured in MN/m^2^ and represented with a heat map, whereby red indicates areas with peak stress. **(A,B)** Lateral stress test of I-beam and triangular truss EIB holder design. **(C–E)** Rotational stress tests of anti-torque guide rails with single post, double post, and reinforced double post designs.

We first used FEA to compare and validate two alternate designs for the EIB holder, a pair of 0.06 g I-beams and a pair of 0.09 g triangular trusses (**Figures 2A,B**). We assessed EIB holder performance for these designs under realistic loads of 1 N during both lateral compression analogous to the stresses associated with adjusting, and vertical compression similar to connecting a headstage. We found that under lateral loads, the lightweight I-beam design resulted in superior performance compared to the triangular truss (10.02 N/m^2∗^10^6^ peak stress and 0.202 mm displacement compared to 11.66 N/m^2∗^10^6^ and 0.272 mm, respectively), however, had slightly inferior performance during vertical compression (5.70 N/m^2∗^10^6^ peak stress and 0.149 mm displacement compared to 5.06 N/m^2∗^10^6^ and 0.097 mm). Since the sides of the EIB holders are the more frequent point of failure during assembly, lateral performance is more critical.

We then used FEA to design and optimize the anti-torque guide rails that secure the top-pieces and maintain electrode alignment with the microscrew. A key weakness in earlier iterations of the SLIQ hyperdrive was substantial top piece movement during driving, which correlates with electrode targeting accuracy. The purpose of the anti-torque guide rail is to reduce top piece lateral rotation around the microscrew, which is a proxy for movement of the electrode’s fixation point on the opposite side of the top piece. Therefore to improve top piece stability, we tested three new designs of the guide rail, a single post, a double post, and a reinforced double post (**Figures 2C–E**). Realistic loads of 2 N were applied 0.5 mm from the axis of rotation (the screw head radius), corresponding to 1 mN^∗^m, which is the approximate torque applied when driving the microscrew. Peak stress was 26.6 N/m^2∗^10^6^, 16.2 N/m^2∗^10^6^, and 9.1 N/m^2∗^10^6^ for the single post, double post, and reinforced double post, while maximum displacement was 1.37, 0.68, and 0.27 mm, respectively. Together, these results indicate that the reinforced double post design of the guide rail will best maintain electrode position.

### Empirical Evaluation of Microdrive Stability

**FIGURE 3:**
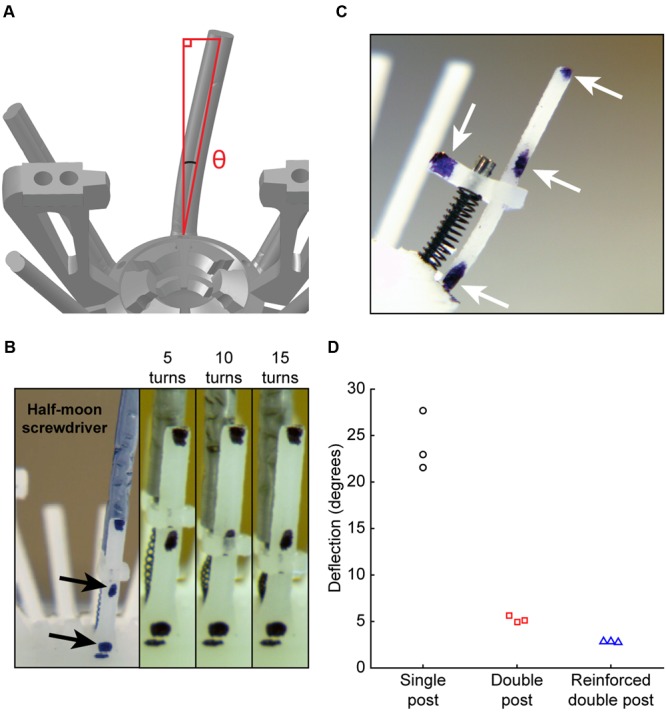
**Empirical evaluation of microdrive stability. (A)** Schematic of angle measurement for top piece lateral stability. **(B)** Side view of microdrive, including top piece, drive spring, microscrew, and anti-torque rail. Arrows denote reference markers. **(C)** Experimental preparation for measurement of top piece stability. Left: wide angle view showing screwdriver. Right: zoomed views for video measurement of lateral displacement during driving, showing measurements at 5, 10, and 15 turns. **(D)** Guide rail deflection angle of each design after driving for 15 turns.

The key feature for microdrive stability during adjusting is lateral movement of the anti-torque guide rail at the top piece (**Figure 3A**). To empirically test the suggestion from FEA that a guide rail with a reinforced double post would improve microdrive stability, we next assembled three implants, one for each guide rail design. For video analysis, each guide rail was marked with reference points (**Figure 3B**, arrows). We then adjusted the microdrive by 15 full turns with a half-moon screwdriver and observed guide rail displacement relative to a stationary contact point at the base of the guide rail (**Figure 3C**, arrows). We found that the single post design resulted in significantly higher lateral movement (24.1 ± 3.2°) than either the double post (5.23 ± 0.35°) or reinforced double post (2.80 ± 0.36°) designs [**Figure 3D**, one-way between groups ANOVA, *F*_(2,6)_ = 118.1, *p* = 0.000015, η^2^ = 0.98, *post hoc* single post to double post and reinforced double posts *p* = 0.000041 and 0.000020], while the lateral movement for the double post and reinforced double post designs were not different from each other (*post hoc p* = 0.32). This reduction in lateral movement for double post designs, confirming FEA predictions, likely results from a combination of increased guide rail strength and increased rotational friction of the top piece in contact with the guide rail.

### Empirical Evaluation of Electrode Placement Accuracy

**FIGURE 4:**
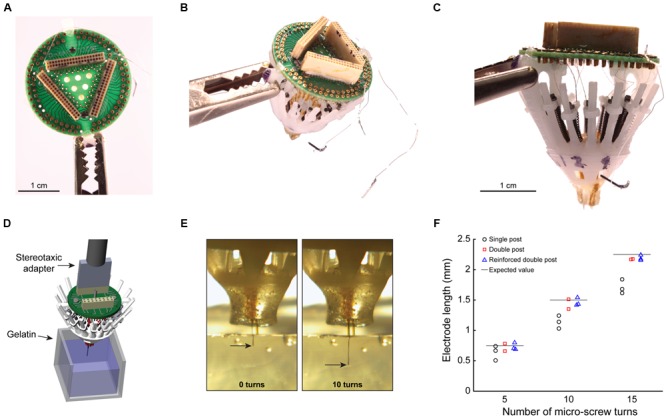
**Empirical evaluation of electrode placement accuracy. (A)** Top view of a completely loaded SLIQ hyperdrive (sans shield), showing a 96 channel EIB and Omnetics connectors. **(B)** Three-quarter view from above, with ground and EEG wires visible. **(C)** Side view, showing individually adjustable microdrives loaded with tetrodes, which protrude from the bottom piece. The anti-torque rails shown are the reinforced double pole design. **(D)** Illustration of the experimental setup to measure electrode displacement during driving. The SLIQ hyperdrive was attached to a stereotaxic via a custom adapter. **(E)** Measurement of electrode placement during driving. **(F)** Quantification of electrode length per turn of 15 full turns of the microscrew.

**FIGURE 5:**
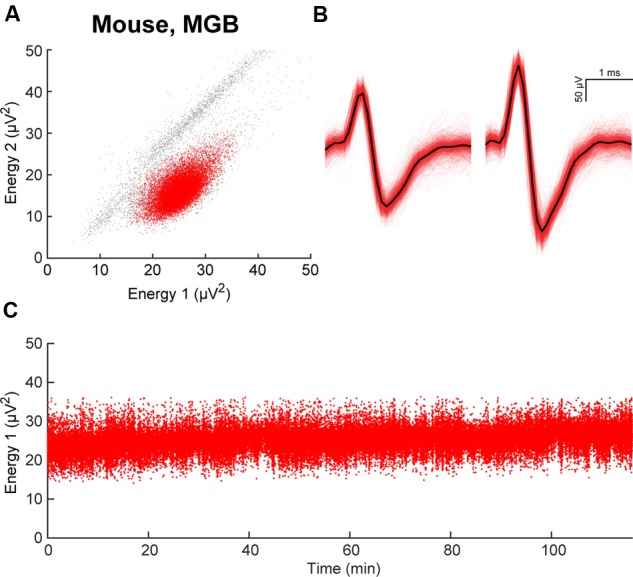
**Stability of electrode placement in awake animals. (A)** Example of clustering in energy space, responses recorded from an electrode array implanted in mouse primary auditory thalamus (MGB). A single cluster is identified in red. **(B)** Waveforms of unit isolated in **(A)**. **(C)** Energy from electrode 1 during a 2-h recording session demonstrates stable electrode placement *in vivo*.

To describe whether the increased mechanical stability of the double post guide rail designs translates into improved electrode placement accuracy, we assembled three SLIQ hyperdrives for evaluation (**Figures 4A–C**, reinforced double post design shown). We then fixed the implants a stereotaxic, and drove individual twisted tetrodes into a translucent brain mimicking material (**Figure 4D**). Due to microscrew thread properties, expected electrode displacement per full turn was 0.15 mm, or 2.25 mm over 15 turns. Electrode penetration was video recorded and measured with LoggerPro Image Analysis (**Figure 4E**). We next compared deviations from the expected value at 5, 10, and 15 turns across guide rail design (**Figure 4F**). Absolute value of the error was highest for the single post design (0.339 ± 0.114 mm) and lower for the double post and reinforced double post design (0.073 ± 0.045 mm and 0.059 ± 0.024 mm, respectively), which were not different from each other [3 × 3 mixed repeated measures ANOVA, *F*_(2.17,5.44)_ = 8.00, *p* = 0.023, η^2^ = 0.76; *post hoc* test between double post designs, *p* = 0.96]. Thus, the double post designs of the anti-torque guide rail substantially improve electrode placement accuracy compared to the single post iteration, most likely through the prevention of top-piece rotation around the guide rail. Once implanted, the SLIQ drive allows for precise targeting and adjustment needed to effectively isolate single unit activity in awake animals (**Figures 5A,B**). The reinforced double post design of the microdrive allows for stable unit isolation across multi-hour recording sessions (**Figure 5C**).

### Use of the SLIQ Hyperdrive to Target Neurons across a Range of Experimental Designs

**FIGURE 6:**
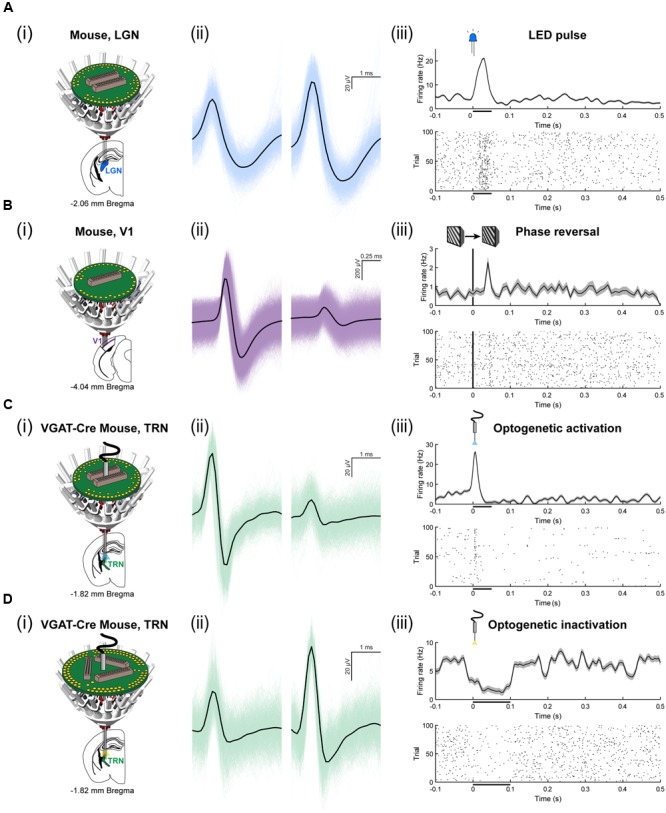
**Use of the SLIQ hyperdrive to target neurons in mice across a range of experimental designs. (A)** (i) Recording schematic for mouse LGN, blue, using a 64 channel EIB. (ii) An isolated, clustered unit, and (iii) visual stimulus-evoked response to 50 ms, LED pulse. **(B)** Recording schematic for mouse layer IV of binocular V1, purple, using a 32 channel EIB. (ii) An isolated, clustered unit, and (iii) visual stimulus-evoked response to a phase reversal of sinusoidal gratings. **(C)** Optogenetic recording schematic for mouse TRN, green, neurons labeled with injection of DIO-ChR2-eYFP retrograde lentivirus into MGB, using a 64 channel EIB and an optical fiber. (ii) An isolated, clustered unit, and (iii) laser stimulus-evoked response to a 50 ms laser pulse (473 nm, 8 mW). **(D)** Optogenetic recording schematic for mouse TRN, green, neurons labeled with injection of DIO-eNpHR3.0-eYFP retrograde lentivirus into MGB, using a 96 channel EIB and an optical fiber. (ii) An isolated, clustered unit, and (iii) laser stimulus-evoked response to a 100 ms laser pulse (561 nm, 8 mW).

**FIGURE 7:**
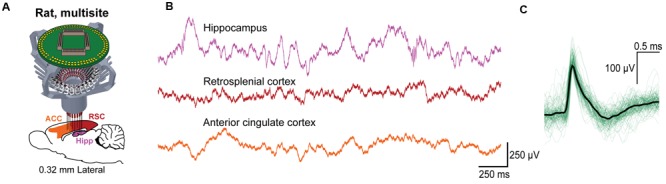
**Use of the SLIQ hyperdrive for multisite recordings in rat. (A)** Schematic for recordings across multiple regions in rat using a 128 channel EIB and a larger, modified drive body. **(B)** Simultaneously recorded signals in hippocampus, retrosplenial cortex, and anterior cingulate cortex, and **(C)** putative hippocampal unit identified on a single electrode.

To assess the ability for the SLIQ hyperdrive to be scaled and modified according to targeting needs, we next designed and assembled a number of implants for a diverse set of experimental preparations. We adjusted the number of necessary microdrives and EIB channels, used a mix of single electrodes, stereotrodes, and tetrodes per microdrive, and incorporated optical fibers for optogenetic tagging in mice. Shown is a representative subset of recordings across multiple brain regions (**Figure 6**). A basic validation of targeting specificity combines precise electrode placement with stimulus-evoked responses in sensory circuits. This is demonstrated by targeting either visual thalamus (lateral geniculate nucleus; LGN) or layer IV of primary visual cortex (V1), and observing firing rate changes when temporally precise sensory stimuli are presented, such as a light pulse or sinusoidal gratings with a phase reversal (**Figures 6A,B**). Next, to specifically target inhibitory (VGAT-positive) neurons in the TRN that project to auditory thalamus (medial geniculate body; MGB), we used the VGAT-Cre mouse, in which Cre recombinase is expressed under the vesicular GABA (γ-aminobutyric acid) transporter promoter. We then selectively labeled MGB-projecting TRN neurons by injecting retrograde lentiviruses (either DIO-ChR2-eYFP or DIO-eNpHR3.0-eYFP) into the MGB. Auditory TRN neurons were then identified as units that, in addition to stimulus-evoked responses, also respond to brief laser pulses delivered by an optical fiber (**Figures 6C,D**). Finally, we designed a reinforced implant with four EIB holders for use in rats to acquire multisite recordings (**Figure 7A**). Shown are simultaneous local field potentials in the hippocampus, retrosplenial cortex, and anterior cingulate cortex (**Figure 7B**), and a putative unit recorded from single electrodes (**Figure 7C**).

## Discussion

Historically, the weight and materials of implants with independently adjustable electrodes limited their use to larger rodents (Yamamoto and Wilson, 2008; Headley et al., 2015), and technical limitations have restricted the use of these approaches in small but genetically tractable rodent models. Although lightweight adjustable drives suitable for recordings in mice have previously been developed (Battaglia et al., 2009; Voigts et al., 2013), these systems have limited design flexibility and are not easily scaled for multi-site recording or use in larger species. The flexDrive (Voigts et al., 2013) provided a solution for use of independently adjustable electrode arrays in mice with ultra-light weight components, however, these implants were necessarily less structurally sound than their predecessors, and the drive design could not be easily adapted to new preparations.

Here, we present a lightweight, independently adjustable electrode array platform that readily integrates optogenetic manipulation for use in small rodents during freely moving behavior. The SLIQ hyperdrive is easier to assemble and customize than existing implants, with improved electrode placement accuracy. We designed this platform to be readily modified to accommodate experimental needs, including a microdrive that can be added iteratively up to 32 microdrives per implant, and compatible EIBs with 32–128 channels designed to accommodate several optical fibers. We then present microdrive validation tools, including FEA and a novel *in situ* experimental setup, to allow experimenters to assess top piece stability and electrode placement accuracy following custom modifications. Finally, we demonstrate use of the implant with experimental preparations in both mice and rats, from multiunit recordings during optogenetic manipulation to multisite local field potential recordings.

Use of the SLIQ hyperdrive during freely moving behavior in transgenic mice has enabled substantial insights into the functional circuitry of deep brain structures such as the thalamus (Halassa et al., 2014; Chen Z. et al., 2015; Wimmer et al., 2015; Wells et al., 2016). Importantly, this implant can incorporate fiber photometry for measurements of local inhibition (Wells et al., 2016) or terminal activity (Gunaydin et al., 2014). To further improve design and fabrication of future implants, experimenters may choose to use third-party vendors for high quality printing of components and assess electrode placement accuracy using a range of available 3D printing materials, as some parameters are prone to variations with current printing techniques. To improve measurement precision when assessing electrode placement, one can embed both a high-resolution distance tape and beads of consistent diameter into the brain mimicking material. Lastly, while narrow (<100 μm core) fibers can be adjusted with microdrives, this implant would benefit from a microdrive mechanism for adjusting the depth of large-diamater (200–400 μm core) optical fibers.

## Author Contributions

LL performed experiments, analyzed data, and contributed to the writing. SO analyzed data, created the figures, and contributed to the writing. JK performed experiments. LS performed experiments, analyzed data, and contributed to the writing. RK performed experiments and analyzed data. MR performed experiments and analyzed data. MH supervised the experiments, directed the analysis, and contributed to the writing.

## Conflict of Interest Statement

The authors declare that the research was conducted in the absence of any commercial or financial relationships that could be construed as a potential conflict of interest.

## Footnotes

1 http://redishlab.neuroscience.umn.edu/MClust/MClust.html

## Supplementary Material

TABLE S1 **Materials and vendor information for the components used in assembling the SLIQ drive**.
